# Influences of Blood Lactate Levels on Cognitive Domains and Physical Health during a Sports Stress. Brief Review

**DOI:** 10.3390/ijerph17239043

**Published:** 2020-12-04

**Authors:** Marinella Coco, Andrea Buscemi, Tiziana Ramaci, Matej Tusak, Donatella Di Corrado, Vincenzo Perciavalle, Grazia Maugeri, Valentina Perciavalle, Giuseppe Musumeci

**Affiliations:** 1 Department of Biomedical and Biotechnological Sciences, University of Catania, 95123 Catania, Italy; 2 Motor Activity Research Center (CRAM), University of Catania, 95123 Catania, Italy; g.musumeci@unict.it; 3 Horus Social Cooperative, 97100 Ragusa, Italy; andreabuscemi@virgilio.it; 4 Department of Research, Italian Center Studies of Osteopathy, 95100 Catania, Italy; 5 Department of Human and Social Sciences, Kore University, 94100 Enna, Italy; tiziana.ramaci@unikore.it; 6 Head of Department of Social and Humanistic Science in Sport Head of the Laboratory for Sport Psychodiagnostics, University of Liubljana, 1000 Liubljana, Slovenia; matej.tusak@fsp.uni-lj.si; 7 Department of Human and Social Sciences, School of Sport Sciences, Kore University, 94100 Enna, Italy; didinawoody@gmail.com (D.D.C.); perciava@libero.it (V.P.); 8 Department of Biomedical and Biotechnological Sciences, Anatomy, Histology and Movement Sciences Section, School of Medicine, University of Catania, 95123 Catania, Italy; graziamaugeri@unict.it; 9 Department of Educational Sciences, University of Catania, 95100 Catania, Italy; valentinaperciavalle@hotmail.it; 10 Department of Biology, College of Science and Technology, Temple University, Philadelphia, PA 19122, USA

**Keywords:** acute exercise, cognition, physical health, sports stress, blood lactate

## Abstract

The present review aims to examine the effects of high blood lactate levels in healthy adult humans, for instance, after a period of exhaustive exercise, on the functioning of the cerebral cortex. In some of the examined studies, high blood lactate levels were obtained not only through exhaustive exercise but also with an intravenous infusion of lactate while the subject was immobile. This allowed us to exclude the possibility that the observed post-exercise effects were nonspecific (e.g., cortical changes in temperature, acidity, etc.). We observed that, in both experimental conditions, high levels of blood lactate are associated with a worsening of important cognitive domains such as attention or working memory or stress, without gender differences. Moreover, in both experimental conditions, high levels of blood lactate are associated with an improvement of the primary motor area (M1) excitability. Outside the frontal lobe, the use of visual evoked potentials and somatosensory evoked potentials allowed us to observe, in the occipital and parietal lobe respectively, that high levels of blood lactate are associated with an amplitude’s increase and a latency’s reduction of the early components of the evoked responses. In conclusion, significant increases of blood lactate levels could exercise a double-action in the central nervous system (CNS), with a protecting role on primary cortical areas (such as M1, primary visual area, or primary somatosensory cortex), while reducing the efficiency of adjacent regions, such as the supplementary motor area (SMA) or prefrontal cortex. These observations are compatible with the possibility that lactate works in the brain not only as an energy substrate or an angiogenetic factor but also as a true neuromodulator, which can protect from stress. In this review, we will discuss the mechanisms and effects of lactic acid products produced during an anaerobic exercise lactate, focusing on their action at the level of the central nervous system with particular attention to the primary motor, the somatosensory evoked potentials, and the occipital and parietal lobe.

## 1. Introduction

At the beginning of the twentieth century, British physiologists W.M. Fletcher and F.G. Hopkins demonstrated that, in the absence of oxygen, muscles are capable of continuing their contraction by producing lactate [1]. In the beginning, it was thought that the increase in lactate was responsible for muscle fatigue [2]; later, however, this theory was abandoned [3], and it was proposed that lactate could play a protective role by promoting muscle response to nerve impulses [4].

**Figure 1:**
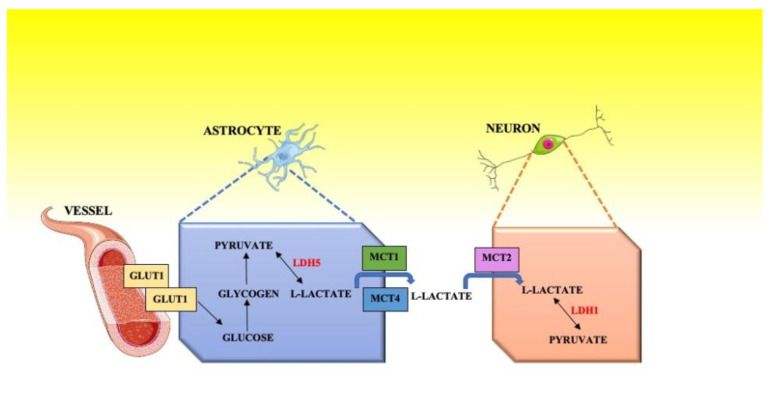
Shuttle model.

Since the 1920s, researchers have observed how lactate diffuses in the blood as well as how its concentration increases rapidly after exhaustive exercise [5]. Geers and Gros [6] demonstrated how blood transports lactate partly dissolved in the plasma and partly in the erythrocytes where it enters both actively and by simple diffusion. First, George A. Brooks [7] and then Pierre Magistretti and Luc Pellerin [8,9] have shown that the nerve tissue not only receives lactate from the blood but is also able to produce it at the level of the astrocytes. Once produced, lactate leaves astrocytes to go into neurons and is used as a useful metabolite; this process has been termed the lactate shuttle. Magistretti and Pellerin show in the theory of the “lactate shuttle” that the production of energy in the central nervous system (CNS) can be divided into two stages: anaerobic glycolysis and aerobic metabolism (Krebs cycle and oxidative phosphorylation); the first transformation process occurs in astrocytes, the second occurs in neurons [10]. Both transformation processes are regulated by the neurotransmitter glutamate, which is released by neurons and absorbed by astrocytes. Glutamate triggers the initiation of anaerobic glycolysis in astrocytes [10] and the production of lactate. The lactate produced is used as the main substrate during brain activation, as described by the astrocyte-neuronal lactate shuttle model (ANLS) proposed by Magistretti and Pellerin; this explains the lower consumption of glucose during stimulation [10]. In the mammalian CNS, and specifically inside the astrocytes, the chemical process takes place that transform glucose into pyruvate, this process is guaranteed by the presence of the enzyme lactate dehydrogenase 5 (LDH5) which catalyzes the conversion of lactate into pyruvate and the reverse reaction [10]. (Figure 1) [9,10].

The uptake of lactate is rapid in astrocytes, expressing low-affinity monocarboxylate transporters (MCTs), i.e., MCT1 and MCT4. In neurons, by contrast, blood lactate levels had little effect on the activity of the high-affinity transporter MCT2 [11]. Exhaustive exercise leads to an increase in the cerebellum of MCT2 expression, thus suggesting that the lactate, by improving the cerebellar metabolism, promotes motor performance [12]. 

In this review, we will discuss the mechanisms and effects of lactic acid products produced during an anaerobic exercise lactate, focusing on its action at the level of the central nervous system with particular attention to the primary motor, the somatosensory evoked potentials, and to the occipital and parietal lobe.

## 2. Materials and Methods

The literature search on the online database was performed using the following databases: EBSCO, MEDLINE, PubMed, Scopus, and Web of Science. No restrictions have been imposed on the date of publication. The study was conducted in accordance with the Declaration of Helsinki. The data relate to studies relating to the influences of blood lactate levels on cognitive domains and physical health during sports stress. We excluded papers that were only available in full in peer-reviewed journals.

## 3. Lactate as a Possible Neuromodulator

Another question that arises in this context is whether lactate is the only a source of energy in the CNS, as previously suggested, or whether it also performs neuromodulatory functions?

One important piece of support for this hypothesis was the identification in the adipocytes of a protein (G protein-coupled receptor 81 or GPR81, also called hydroxycarboxylic acid receptor 1 or HCAR1) capable of functioning as a receptor for lactate which, in this way, exerts an anti-lipolytic action [13]. In 2015, the lactate receptor GPR81 (or HCAR1) was located in excitatory synapses of the CNS [14]. 

The authors conclude that lactate can act as a volume transmitter through the activation of HCAR. in this way, neuronal activity, cerebral blood flow, energy metabolism, and the availability of the energy substrate, including the response to glucose and glycogen savings, are linked by lactate. HCAR1 can help optimize the concentration of cyclic adenosine monophosphate (cyclic AMP or cAMP) [14].

**Figure 2:**
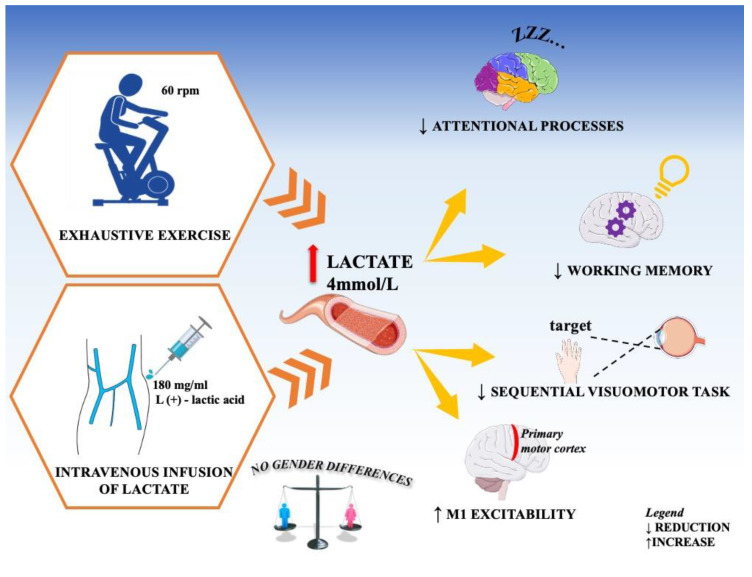
General diagram of the effects of lactic acid. When blood lactate levels exceed 4 mmol/L, this corresponds to the onset of blood lactate accumulation (OBLA). OBLA indicates the passage from aerobic exercise to anaerobic exercise.

As a result, over the past ten years, numerous studies were carried out with the purpose of evaluating whether an increase of blood lactate is able to modify brain functions in humans by acting as a neuromodulator. Below, we briefly review the literature on the relationship between blood lactate levels and cognition as well as the effects of high lactate levels on cortical excitability. Overall findings are summarized in Figure 2.

## 4. Studies on Cognition

Coco and collaborators [15] wrote the first study to discover the possible influences of high blood lactate on the cognitive processes of attention, considering attentional processes as a controlled system of information processing and decision-making activities (*European Journal of Applied Physiology*, 2004) [16]. This controlled attention process includes aspects such as intensity (vigilance and sustained attention) and selectivity (broad, focused, and divided attention) (*European Journal of Applied Physiology*, 2004) [17].

The term “broad attention” means the ability to evaluate a large amount of information, while the term “selective attention” means the ability to discriminate between stimuli and the selection of a small number of stimuli despite the presence of distracting stimuli [17]. 

This cognitive domain includes aspects of attention such as intensity (sustained attention and alertness) and selectivity (divided, focused, and broad attention). In particular, the ability to analyze a large amount of information is a characteristic of broad attention, while selective attention permits one to discriminate between stimuli and to select some of them even in the presence of distracting stimuli [18]. 

In this study, the authors obtained the increase of blood lactate in two ways:

With an exhaustive exercise, e.g., by executing an incremental cycling test on a cycle ergometer (Monark, Sweden), at a pedaling rate of 60 rpm. Each participant began to pedal without resistance, and then, every 3 min, the resistance increased by 30 watts, and it continued until the subject could no longer maintain the rhythm of 60 rpm [6].

Since as a consequence of an intense physical activity many variables, in addition to blood lactate levels, can vary in an organism (e.g., cytokines, hormones, temperatures, pH, etc.), the effects induced by an intravenous infusion of lactate were evaluated while the subject was at absolute rest. A sterile lactate solution (concentration: 2 mEq/mL) containing a mixture of 180 mg/mL of L (+)—lactic acid and 80 mg/mL of NaOH, at pH of 6.5–8.0 (Monico S.p.A., Venice, Italy) was infused in the cubital vein of the arm at a dose of 3 mg/kg in 1 min. During the infusion, the subject was comfortably seated in an armchair. 

The validated Italian version of the attention and concentration tasks (ACT, Erickson) was used [19]. The ACT comprises several tasks, specifically designed for the assessment of different attentional capabilities with a personal computer. Two of the ACT tests were utilized, the first for evaluation of reaction time (RT), for the intensity of attention, and the second for divided attention, for the selectivity of attention [20]. 

The levels of blood lactate were assessed through a portable lactate analyzer (Lactate Pro; FaCT Canada Consulting Ltd; Arkray Inc., Kyoto, Japan).), as it has been shown that this lactate analyzer has both good sensitivity and reliability [21].

Measures were performed before the exhaustive exercise or the lactate infusion, as well as 5 and 10 min after its end.

It was seen that, after intense physical exercise, the increase in blood lactate (10 mmol/L) is associated with a significant worsening of attentional processes (*p* < 0.001 ***). The same result occurs after an infusion of lactate in the blood which increases its blood values (4.5 mmol/L), it was therefore concluded that the increase in blood lactate levels is in itself capable of interfering with the mechanisms of attention.

Afterwards, a series of experiments were carried out to identify the blood lactate levels that could affect the attentive processes. Perciavalle and colleagues [22,23,24,25,26,27,28,29,30,31] observed that the negative effects on the attentive processes are detected only when blood lactate levels exceed 4 mmol/L, corresponding to the onset of blood lactate accumulation (OBLA) [32]. 

This observation is consistent with findings from previous studies reporting how aerobic physical exercise increases the level of learning-related neurotrophins [32] and accelerates long-term potentiation (LTP)-like plasticity in primary motor area (M1) [32], thus being a potential modulator of motor learning [33,34,35,36].

The effects of high blood lactate levels on another cognitive domain (i.e., working memory; WM) were also assessed on 30 adult subjects equally distributed across sexes using two different protocols [37]. The first protocol focused on the analysis of non-spatial WM, including the use of the self-ordered pointing task (SOPT), which allows for producing and monitoring a sequence of responses [38]. The second protocol focused on the assessment of the motor WM needed to accomplish a motor task, and was an adaptation of the protocol designed by Rottschy and colleague [38]. In this protocol, the increase of blood lactate was obtained with an exhaustive exercise on a cycle ergometer, whereas the levels of blood lactate were measured through a portable lactate analyzer, as previously described. Assessment of both types of WM as well as of blood lactate levels were performed before and after exercise. Another follow up assessment was also completed 15 min after the conclusion of the session.

It was observed that the increase in blood lactate after acute exhaustive exercise (12 mmol/L) correlates with a significant worsening of WM (*p* < 0.001 ***). The increases in test errors, both in attention and memory, were independent of sex.

A 2016 study [39] confirmed this line of research by investigating the relationship between the effects of maximal exercise and elevated blood lactate levels on the performance of a cognitive task, and the relationship with immediate (explicit) (*p* < 0.001 ***) lactate produced (12 mmol/L) and long-term (implicit) lactate produced through an exercise in precision (*p* < 0.001 ***) [39]. Furthermore, with transcranial magnetic stimulation (TMS), on healthy subjects, it was determined that changes in motor performance are associated with changes in excitability of the primary motor area (M1) and supplementary motor area (SMA) [39].

The increase in blood lactate was obtained with an exhaustive exercise on a cycle ergometer, whereas the levels of blood lactate were measured through a portable lactate analyzer (Lactate Pro; FaCT Canada Consulting Ltd.), as previously described. Excitability of cerebral cortex was evaluated by using transcortical magnetic stimulation (TMS). Magnetic stimuli produced through a Magstim 200 stimulator (Magstim Co., Dyfed, UK) were delivered by means of a figure-of-eight coil positioned at the primary motor cortex. The coil was placed in the best position (hot spot) for obtaining a motor-evoked potential (MEP) from a muscle in the contralateral arm. Once the hot spot was identified, the lowest stimulation intensity at which MEPs were produced in at least 5 of 10 consecutive trials was considered the resting motor threshold (RMT). The recruitment curve of MEPs was achieved by sending TMS at 10% increases of intensity between 100 and 140% of the RMT. Usually, six stimuli were randomly delivered at every level of intensity, at intervals between 4 and 6 s. In this study, “to maintain constant the position of coil throughout the entire TMS session, a neuronavigation system (Softaxic Optic system 2.0, E.M.S. srl Company, Bologna, Italy), equipped with an optical tracking system NDI Polaris Vicra (NDI International, Waterloo, ON, Canada), was used” (Marinella Coco, 2016) [39]. All assessments were performed before the exhaustive exercise, as well as at its conclusion and 15 min after its end. 

A sharp increase in blood lactate levels after an acute exhaustive exercise (mean value 12.0 to 12.2 mmol/L) is associated with a significant worsening of immediate (defined as the explicit trial; *p* < 0.001 ****) as well as delayed (defined as the implicit trial; *p* < 0.001 ****, i.e., highly significant) reproduction of sequential visuomotor task paradigm, without gender differences. [39].

Moreover, after an exhaustive exercise, a variation of excitability of both M1 and SMA was observed. In particular, an increase in the excitability of M1 was detected, whereas a decrease in SMA was observed, without gender differences (concerning the relationship between cortical excitability and gender, see below). Opposite effects on the excitability of adjacent cortical areas lead us to exclude the possibility that the observed effects are nonspecific, due, for example, to a post-exercise change of cortical pH, temperature etc. [2].

## 5. Studies with Transcranial Magnetic Stimulation

The first study [21,40] performed to evaluate the effects of high blood lactate levels on the excitability of the cerebral cortex found that increases in blood lactate levels and decreases in motor thresholds were significant.

Even in subjects who agreed to undergo lactate infusion to increase blood lactate, the motor threshold is decreased. Furthermore, after infusion of a lactate solution, statistical analysis revealed a significant correlation between increases in blood lactate and decreases in the threshold. The authors concluded that increased blood lactate is able to affect M1 excitability, worsening performance.

The next step was to evaluate possible differences in gender in the observed effects [22]. Forty-one adult athletes volunteered for this research (20 women and 21 men); women did not use oral contraceptives and were analyzed in the midluteal phase of the menstrual cycle. The study compared cortical excitability and blood lactate levels in men and women, evaluated at rest (pre-exercise), at the conclusion of the exhaustive exercise (end of exercise), 5 min after its end, and finally 10 min after its conclusion. While men and women did not differ regarding blood lactate levels before and after exercise, women showed a significantly greater increase in excitability of M1 (*p* < 0.05) after exercise than their male counterparts. Based on these results, it is possible to conclude that women’s cortex of M1 possesses a greater sensitivity for lactate than men.

The next step was to study whether the positive effect of lactate on neuronal excitability is specific for the motor cortex or it is also present on neurons of subcortical structures. To answer this question, the excitability of spinal motoneurons and that of brainstem structures were analyzed by means of the H reflex and blink reflex, respectively [23,41].

In both these studies, it was observed that a sharp increase of blood lactate levels is associated with a significant rise in the excitability of spinal and brainstem neurons. However, as an increase in lactate induced by an infusion in a subject at rest causes no change in excitability either at the spinal level or at the brainstem level, it was concluded that the raise of blood lactate levels induced by an exhaustive exercise is not per se capable of increasing the excitability of subcortical structures.

Because in this first series of experiments on the evaluation of cortical excitability, the exercise involved the legs but the hot spot identified with TMS controlled contralateral hand muscles, a further step was to compare the effects of blood lactate levels on cortical excitability after an exhaustive exercise involving the hand [22]. Eight healthy right-handed male subjects were seated in an armchair with a strain gauge dynamometer G100 (Biometrics Ltd., Gwent, UK) held in the right hand, while the left hand was relaxed in the prone position. Each of the subjects had to grip the dynamometer, in order to produce a tonic contraction of the flexor carpi radialis muscle. Moreover, each of them had to exert the maximal isometric grip effort for 5 s in 3 successive trials to assess the maximal voluntary grip. 

It was observed that a fatiguing handgrip exercise causes a small but statistically significant rise in blood lactate levels. Moreover, it was detected that the excitability of M1 decreased immediately after exercise and 5 min after its conclusion if compared to pre-exercise levels. In these conditions, it was observed that the subjects who, after the exercise, had the highest blood lactate levels, exhibited less reduction in MEP amplitude. Ten minutes after the end of the exhaustive exercise, the positive association between blood lactate levels and MEP amplitude vanished. The authors concluded that after a very intense physical exercise, lactate might be able to delay the onset of fatigue not only by preserving the excitability of muscle [4] but also by improving the excitability of M1 [39].

Moscatelli and colleagues [42] confirmed these results, observing that high levels of blood lactate increase the excitability of the primary motor cortex more in athletes than in non-athletes. Similarly, O’Leary et al. [43] also detected that an exhaustive exercise reduces long-interval intracortical inhibition.

## 6. Studies with Sensory Evoked Potentials

Studies have been conducted on the effects of high levels of blood lactate on cortical areas located outside the frontal lobe by using sensory evoked potential. 

Studies on the effects of high blood lactate levels on cortical areas located outside the frontal lobe were conducted using sensory evoked potential. The increase in blood lactate values, obtained with exhaustive exercise (12.9 mmol/L) or with an intravenous infusion of a lactate solution (5.18 mmol/L), is associated with significant changes in the latency of two components of the visual evoked potentials: P100 and N145 (*p* < 0.05) [21,30]. In particular, it was observed that the latency of P100 displays a significant reduction after the exercise (0.0341 *), whereas the latency of N145 exhibits an increase of latency 10 min after the end of the exercise (0.0255 *). There is general consensus on the idea that the P100 is generated in the striate cortex of the occipital lobe, whereas the N145 component in the extrastriate visual cortex [44]; Coco et al. [28] concluded that an increase in blood lactate seems to be able to improve the conduction time between the retina and striate cortex, while it seems to produce a slowdown of communications between the striate cortex and the extrastriate areas.

The authors [45] observed similar results, analyzing the effects of high blood lactate levels of lower limb somatosensory evoked potential (SEP), specifically focusing on the amplitude and latency of P1, N1, P2, and N2 components. The authors [45] observed similar results analyzing the high levels of lactate in the blood effects of the somatosensory evoked potential of the lower limbs (SEP), focusing in particular on the scale and latency of components P1, N1, P2 and N2. After intense exercise, a significant reduction in breadth P2-N2 (*p* = 0.0164 *) and the latency of the components P1 and N1 (*p* = 0.1143) is observed, whereas there was a significant increase in latency of N2 (*p* < 0.0001****). The results obtained suggest that lactate in the blood can play a protective effect against fatigue, at the primary level cortical areas (such as S1), reducing the efficiency of the adjacent areas (such as S2). 

## 7. Limitation and Future Proposal

A limitation of this paper is not having data collected for measuring invasive of lactate in humans. It would be interesting to perform the same search using more detailed investigations, such as electroencephalography (EEG).

## 8. Conclusions

In conclusion, lactate is no longer considered only as a metabolic waste product, it has been observed that it plays a role at the level of the central nervous system, both directly and indirectly. Research shows that lactate is an important energy substrate in astrocytes, and at the same time plays a protective or modulatory role at the level of primary cortical areas (such as M1, V1 or S1) [46,47,48,49,50,51].

The studies conducted to date will encourage the possibility that lactate works in the brain not only as an energy substrate or modulatory role at the level of primary cortical areas but also as a true neuromodulator [52,53,54].
